# Hepatitis C Virus Infection in Guinea-Bissau: A Sexually Transmitted Genotype 2 with Parenteral Amplification?

**DOI:** 10.1371/journal.pone.0000372

**Published:** 2007-04-18

**Authors:** Mireille Plamondon, Annie-Claude Labbé, Eric Frost, Sylvie Deslandes, Alfredo Claudino Alves, Nathalie Bastien, Jacques Pepin

**Affiliations:** 1 Department of Microbiology and Infectious Diseases and Centre for International Health, University of Sherbrooke, Sherbrooke, Québec, Canada; 2 Hôpital Maisonneuve-Rosemont, Montréal, Québec, Canada; 3 Hopital Simao Mendes, Bissau, Guinea-Bissau; University of Liverpool, United Kingdom

## Abstract

**Background:**

Sub-Saharan Africa is the continent with the highest prevalence of Hepatitis C virus (HCV) infection. Genotype 2 HCV is thought to have originated from West Africa several hundred years ago. Mechanisms of transmission remain poorly understood.

**Methodology/Principal Findings:**

To delineate mechanisms for HCV transmission in West Africa, we conducted a cross-sectional survey of individuals aged ≥50 years in Bissau, Guinea-Bissau. Dried blood spots were obtained for HCV serology and PCR amplification. Prevalence of HCV was 4.4% (47/1066) among women and 5.0% (27/544) among men. In multivariate analysis, the independent risk factors for HCV infection were age (baseline: 50–59 y; 60–69 y, adjusted odds ratio [AOR]: 1.67, 95% CI: 0.91–3.06; ≥70 y, AOR: 3.47, 95% CI: 1.89–6.39), belonging to the Papel, Mancanha, Balanta or Mandjako ethnic groups (AOR: 2.45, 95% CI:1.32–4.53), originating from the Biombo, Cacheu or Oio regions north of Bissau (AOR: 4.16, 95% CI: 1.18–14.73) and having bought or sold sexual services (AOR: 3.60, 95% CI: 1.88–6.89). Of 57 isolates that could be genotyped, 56 were genotype 2.

**Conclusions:**

Our results suggest that transmission of HCV genotype 2 in West Africa occurs through sexual intercourse. In specific locations and subpopulations, medical interventions may have amplified transmission parenterally.

## Introduction

Molecular analyses of Hepatitis C virus (HCV) revealed that the onset and location of their emergence, and patterns of subsequent transmission, varied between the six viral genotypes [1], [2]. Genotype 6, the most ancient, emerged ≈700 years ago in Asia, while genotype 4 did so ≈300 years ago in Africa or the Middle East [1]. Genotype 1b emerged between 1880–1920 in the United States, Brazil and Asia and its dissemination, associated with transfusions and hemodialysis, is slowing down [2]. Genotype 1a emerged in the United States and Brazil ≈1940–50 with rapid exponential growth; its transmission being strongly linked to injection drug use, it continues to expand [1], [2]. In contrast with Central Africa, genotype 2 displays a high diversity in West Africa, from where it presumably originated [3]–[9].[Fig pone-0000372-g001]

**Figure 1 pone-0000372-g001:**
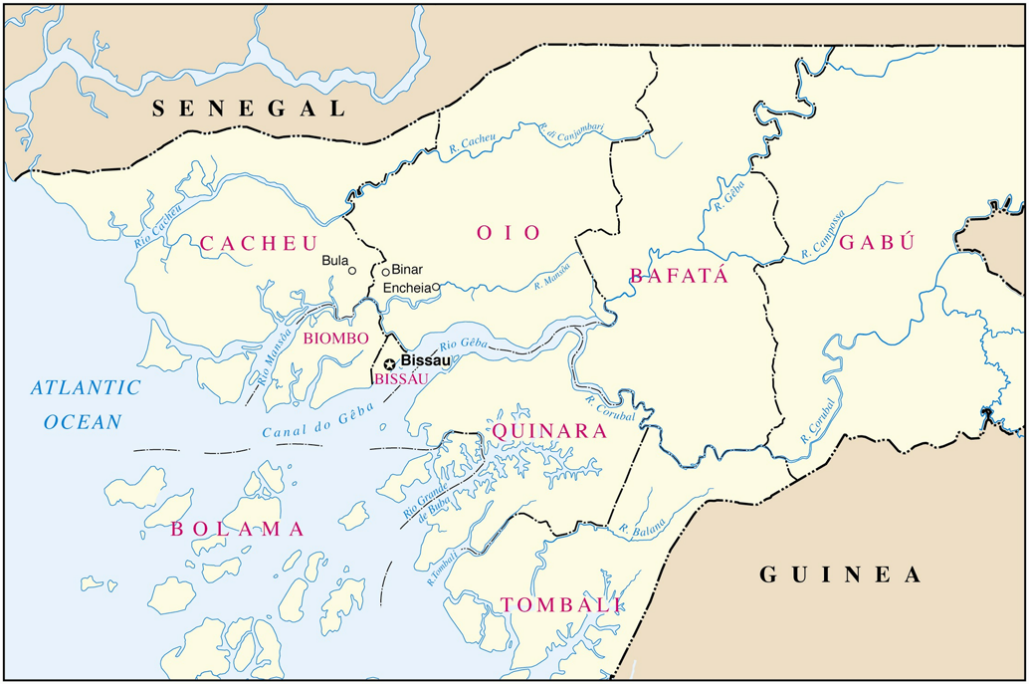
Map of Guinea-Bissau.

Sub-Saharan Africa is the continent with the highest HCV prevalence, 6.0% of adults being HCV-positive in Central Africa, 2.4% in West Africa and 1.6% in Southern/East Africa [10]. It is assumed that unsafe injections, transfusions and other medical or traditional practices explain most of HCV transmission [10], by analogy with Egypt where a massive genotype 4 epidemic resulted from nationwide campaigns using intravenous drugs for the control of schistosomiasis [11], [12]. However, this preponderance of iatrogenic transmission remains unproven in a continent where, apart from patients with sickle cell anemia [6], few receive multiple transfusions, and where HCV is rare among children [10], recipients of multiple injections. Furthermore, HCV transmission through occupational parenteral exposure is relatively ineffective (≈0.5%), except from a hollow needle placed in the vein or artery of a viremic source patient [13], [14]. HCV heterosexual and perinatal transmission is uncommon in developed countries [15]–[17], but modes of transmission might vary according to viral genotypes and genetic background of populations. There is little information concerning sexual transmission of HCV in sub-Saharan Africa, where most studies merely presented prevalence [10]. We sought to identify risk factors for HCV infection as part of a study of the epidemiology of HIV-2 among individuals aged ≥50 years living in Bissau, the capital city of Guinea-Bissau [18].

## Methods

### Data collection

The study had been ethically approved by the Guinea-Bissau ministry of health and the institutional review board of the Centre Hospitalier Universitaire de Sherbrooke. Inclusion criteria were age ≥50 years and willingness to give consent. Exclusion criteria were the presence of dementia or inability to speak one of the several languages spoken by the interviewers. Participants were recruited from the following areas of Bissau: Pluba, Antula, Santa Luzia, Reno, Cupelom, Luanda, Penha, Pefine, Calequir, Bairro Militar, Amedalai and Rossio. From January to March 2005, compounds were visited one by one to identify individuals aged ≥50 years, who were then asked to participate. If a verbal consent was obtained, a questionnaire was administered in a private setting, generally by an interviewer of the same sex as the respondent. Data were gathered on socio-demographic characteristics, past medical histories, and a limited number of questions on sexual behavior. For participants unable to provide their exact age, this was estimated based on historical events widely known in the country. A finger prick was performed and capillary blood deposited on a filter paper. No names were recorded on the questionnaires and filter papers, identified only by a study number.

### Serological assays

Blood samples were blotted onto two Whatman no. 3 filter papers, and allowed to air dry. Samples were stored in plastic zip-closure bags at 4°C for 1–2 months and transported to Canada for testing. All samples were screened for HCV antibodies using Detect-HCV v.3 (Adaltis, Montreal, Canada). A 6 mm dried blood spot (DBS) was punched and placed into 250 µl of the specimen diluent provided with the test kit, in a 96-well plate. The plate was incubated on a shaking platform at room temperature for 30 minutes, and then overnight at 4°C. The eluted sample (200 µl) was used to perform all other steps according to the manufacturer's instructions. Non-reactive samples were considered HCV-seronegative while reactive samples were further tested with both Ortho HCV 3.0 ELISA Test (Ortho-Clinical Diagnostics, Raritan, New Jersey) and Monolisa Anti-HCV Plus Version 2 (Bio-Rad, Marnes-la-Coquette, France). For each test kit, two 6 mm DBS were eluted in respectively 250 µl and 175 µl of the provided specimen diluent (with the same incubation protocol as for Detect-HCV). For Ortho, 170 µl of eluted sample was added to in each reaction well containing 50 µl of specimen diluant, while for Monolisa, 100 µl of eluted sample was used, without adding more specimen diluent. Dually non-reactive samples (Detect-HCV+/Ortho-/Monolisa-) were considered HCV-negative. Repeatedly reactive samples (with all three ELISA tests) were considered HCV positive, while discordant results samples were further tested by INNO-LIA HCV Score (Innogenetics, Ghent, Belgium), using 1 ml of eluted sample (obtained from two 6mm DBS in 1.2 ml of INNO-LIA sample diluent), and the result of the INNO-LIA was considered as definitive. HIV serology was performed using Detect-HIV (Adaltis) as the initial test, followed by Genie II HIV-1/HIV-2 (Bio-Rad) and INNO-LIA HIV I/II Score (Innogenetics) as previously described [18].

### Genotyping

For genotyping, PCR amplification of the samples considered to be HCV seropositive was attempted using the NS5B region. RNA was extracted from 8 circles of 3 mm punched from DBS in 400 µl of Trizol containing 60 ng of polyA (Invitrogen, Burlington, On, Canada) and re-suspended in 30 µl of RNase free water. Reverse transcription and an external amplification cycle were performed with 5 µl of RNA extract and primers EF101F TTCTCGTATGATACCCGCTGYTTTGA and HCVNS5Rnb TACCTGGTCATAGCCTCCGTGAAGGCTC in a final volume of 20 µl C. therm. Polymerase for One-Step RT-PCR System (Roche, Laval, Qc, Canada) for 30 min at 60°C; 2 min at 94°C; then 40 cycles of 94°C for 15 sec, 50°C for 45 sec and 72°C for 1 min or 6 min in the last cycle. One µl of the RT-PCR product was amplified in nested PCR under the same amplification conditions using primers HCVNS5F2p (TATGATACCCGCTGCTTTGACTC,G/I,AC), HCVNS5R2c (CTGGTCATAGCCTCCGTGAAGGCTCTCAGG) and HCVNS5R2d (CTGGTCATAGCCTCCGTGAAGGCTCGTAGG). Amplification was monitored by agarose gel electrophoresis. Amplicons were sequenced using the HCVNS5F2p primer by Genome Quebec (Montreal, Qc, Canada). Each sequence was analyzed by HCV-BLAST (http://hcv.lanl.gov/content/hcv-db/BASIC_BLAST/basic_blast.html) to define the HCV genotype by the closest matching sequences. Samples were also tested for the 5′ non-coding region of HCV using the Amplicor Hepatitis C Virus Test, version 2.0 (Roche) after extracting RNA from two 3 mm DBS using the Charge Switch total RNA kit (Invitrogen, Burlington, On, Canada) and eluting the RNA in 75 µl of elution buffer.

### Statistical analysis

Data were analyzed with Stata 8.0. Proportions were compared with the χ^2^ test, or Fisher's exact test when an expected cell number was less than five. Variables significantly associated with HCV in univariate analysis were tested in logistic regression models built up sequentially, starting with the variable most strongly associated with the outcome and continuing until no other variable reached significance. When the final model was reached, each variable was dropped in turn to assess its effect by the likelihood ratio test. We kept in the final model variables that significantly enhanced the fit at the p<0.05 level.

## Results

Out of 1615 specimens tested, five were considered indeterminate and will not be considered further. Prevalence of HCV infection was 5.1% (69/1347) among the HIV-seronegative participants, 2.5% (5/204) among those infected with HIV-2 and 0% (0/48) among those infected with HIV-1 (p = 0.07).

In Table 1 are shown risk factors for HCV infection amongst all participants (n = 1610). HCV prevalence was 4.4% among women and 5.0% among men (p = 0.76). HCV prevalence increased with age, but did not vary according to marital status. HCV prevalence varied significantly according to ethnic groups, which could be dichotomized into high-risk (Papel, Mancanha, Balanta and Mandjako) and low-risk (all others) groups. HCV prevalence also varied according to region of origin, being lowest in participants from Bafata and Gabu, in the eastern part of the country, and highest among those from the regions of Biombo, Cacheu and Oio, north of Bissau (map). Within these regions, HCV was much more prevalent (24/155 [15.5%] in women, 7/80 [8.8%] in men, 31/235 [13.2%] overall) among individuals who were born and grew up in a small area around Bula, Enchiea and Binar (map) compared to other participants (19/468 [4.1%], p<0.001). HCV prevalence was significantly higher among participants involved in sex work. Most of this association reflected a higher HCV prevalence among men who admitted having ever paid for sexual services (12/118 [10.2%]) compared to those who did not (15/423 [3.5%], p = 0.005); among women, 2/10 (20%) who admitted having sold sexual services were HCV-infected compared to 44/1050 (4.2%) among those who denied such behavior (p = 0.03). Among women, HCV prevalence was 1.8% (9/503) in those who had been excised, compared to 6.8% (38/561) among those who did not undergo excision of the clitoris (p<0.001). Only 19 men were uncircumcised, none of whom were HCV-infected, while 27/523 (5.2%) circumcised men were HCV seropositive (p = 0.62). HCV prevalence was not higher in participants who reported having had traditional scarifications, having been transfused, having been treated for trypanosomiasis or tuberculosis or having received injections of pentamidine for the chemoprophylaxis of trypanosomiasis.

**Table 1:** Risk factors for Hepatitis C Virus (HCV) infection in univariate analyses.

	HCV positive/Total (%)	Odds ratio (95% CI)
Sex		
Male	27/544 (5.0)	1.00
Female	47/1066 (4.4)	0.88 (0.54–1.43)
Age		
50–59	20/726 (2.8)	1.00
60–69	27/526 (5.1)	1.91 (1.06–3.44)^b^
≥70	27/358 (7.5)	2.88 (1.59–5.21)^a^
Marital status		
Married	40/961 (4.2)	1.00
Divorced/Single	7/101 (6.9)	1.71 (0.75–3.93)
Widowed	27/547 (4.9)	1.20 (0.73–1.97)
Ethnic group		
Others	7/338 (2.1)	1.00
Mandinka	6/282 (2.1)	1.03 (0.34–3.09)
Fula	5/209 (2.4)	1.16 (0.36–3.70)
Papel	13/206 (6.3)	3.19 (1.25–8.12)^b^
Mancanha	9/134 (6.7)	3.40 (1.24–9.34)^b^
Balanta	31/396 (7.8)	4.02 (1.74–9.24)^a^
Mandjako	3/44 (6.8)	3.46 (0.86–13.90)
Region of origin		
Bafata-Gabu	4/243 (1.6)	1.00
Biombo-Cacheu-Oio	50/703 (7.1)	4.58 (1.63–12.80)^b^
Quinara-Tombali-Bolama	9/350 (2.6)	1.58 (0.48–5.18)
Bissau	11/286 (3.8)	2.39 (0.75–7.60)
Outside Guinea-Bissau	0/28 (0)	0.00
Ever bought/sold sex		
No	59/1472 (4.0)	1.00
Yes	14/128 (10.9)	2.94 (1.59–5.43)
Ever had genital ulcer		
No	71/1565 (4.5)	1.00
Yes	2/36 (5.6)	1.24 (0.29–5.25)
Ever lived or traveled outside Guinea-Bissau		
No	44/849 (5.2)	1.00
Yes	29/751 (3.9)	0.74 (0.45–1.19)
Pentamidine prophylaxis for trypanosomiasis		
None	47/1136 (4.1)	1.00
1–4 injections	23/400 (5.8)	1.41 (0.85–2.36)
≥5 injections	2/65 (3.1)	0.74 (0.17–3.10)
Ever received treatment for trypanosomiasis		
No	70/1503 (4.7)	1.00
Yes	4/105 (3.8)	0.81 (0.29–2.27)
Ever received injections for treatment of tuberculosis		
No	70/1544 (4.5)	1.00
Yes	4/63 (6.4)	1.43 (0.50–4.04)
Ever received blood transfusions		
No	65/1419 (4.6)	1.00
Yes	9/187 (4.8)	1.05 (0.52–2.15)
Ever had traditional scarifications		
No	47/1111 (4.2)	1.00
Yes	27/497 (5.4)	1.30 (0.80–2.11)
Vaccination scar		
No	26/369 (7.1)	1.00
Yes	48/1228 (3.9)	0.54 (0.32–0.88)^a^

a p≤0.001

b p≤0.05

**NOTE**. Odds ratios and their 95% confidence intervals (CI) were calculated with Stata 8.0.

In multivariate logistic regression analysis (table 2), the independent risk factors for HCV infection were older age, ethnic group, region of origin and having been involved in sex work. No interaction was found between these variables. None of the parenteral exposures that we measured was associated with HCV infection when analysis was restricted to individuals originating from high-prevalence regions (data not shown). Among women, excision became non-significant after adjustment for ethnicity.

**Table 2:** Risk factors for Hepatitis C Virus (HCV) infection in multivariate analysis.

	Adjusted odds ratio (95% CI)
Age	
50–59	1.00
60–69	1.67 (0.91–3.06)
≥70	3.47 (1.89–6.39)^a^
Ethnic group	
Mandinka, Fula or others	1.00
Papel, Mancanha, Balanta or Mandjako	2.45 (1.32–4.53)^b^
Region of origin	
Bafata-Gabu	1.00
Biombo-Cacheu-Oio	4.16 (1.18–14.73)^b^
Quinara-Tombali-Bolama	1.77 (0.46–6.76)
Bissau	1.95 (0.51–7.54)
Outside Guinea-Bissau	0.00
Ever bought/sold sex	
No	1.00
Yes	3.60 (1.88–6.89)^a^

a p≤0.001

b p≤0.05

**NOTE**. Adjusted odds ratios and their 95% confidence intervals (CI) were calculated with Stata 8.0.

Using the 5′ non-coding region, 65 of 70 (93%) HCV-seropositive samples were PCR positive (there was insufficient sample for the other 4 HCV seropositives). All 25 men were PCR-positive compared to 89% (40/45) of women (p = 0.15). Viremia was detected in 89% (16/18), 92% (24/26) and 96% (25/26) of individuals aged respectively 50–59 y, 60–69 y and ≥70 y (p = 0.65). As the 5′ non-coding region is short and highly conserved, we used amplification of the NS5B region for genotyping. Amplification was successful in 57 samples, 56 of which belonged to genotype 2. The only participant infected with genotype 1 had lived for 4 years in Senegal and Mauritania.

## Discussion

Genotype 2 infected almost all viremic HCV seropositive individuals aged ≥50 years living in Bissau, Guinea-Bissau. This preponderance of genotype 2 was higher than the 59% reported in Guinea, Benin and Burkina Faso [6] and similar to the 87% found in Ghana [7]. In a smaller study in Guinea Conakry, all seven HCV isolates that were characterized belonged to genotype 2 [8]. Several studies revealed a high diversity of genotype 2 in West Africa, indicating an ancient origin [6]–[9]. In Martinique, where three fourths of slaves sent in the 17^th^–18^th^ century came from West Africa, there is a high diversity of genotype 2 suggesting that it was already present in the Bight of Benin and Gold Coast at the height of the slave trade, in contrast to genotype 4, recently introduced into this Caribbean island by intravenous drug users [19], [20]. Among HCV-infected patients in Brazil, genotype 2 is more prevalent in Maranhão, the main destination for slaves exported out of Portuguese Guinea in the 18^th^ century [20], [21]. Such molecular, epidemiological and historical evidence suggests that genotype 2 HCV has been present in West Africa for a few centuries, antedating the development of modern medicine and its potential for parenteral transmission, and implying that another mode of transmission was involved.

Our results suggest that sexual transmission played a role in the dynamics of genotype 2 HCV infection in Guinea-Bissau amongst a generation born before 1955. In Bissau, HCV was more prevalent in individuals involved in transactional sex. Among women, this was based on a very small number of participants. Among men, for whom it may be more socially acceptable to admit having paid for the services of sex workers, the association with prostitution was more robust. In Kinshasa, HCV prevalence was higher in sex workers than pregnant women, but only in those aged >30 years [22]. Among sex workers, prevalence increased with age (21% in the >40 years age group) and duration of prostitution. HCV seropositivity was also associated with a prior transfusion and a positive syphilis serology, suggesting a combination of parenteral and heterosexual transmission [22]. A HCV prevalence higher in sex workers than in the general female adult population was also documented in Niger and Ethiopia [23], [24]. In a nationwide survey in the United States, having had more than 20 life time sexual partners was an independent risk factor for HCV [25]. In Italy, incidence of genotype 1 HCV infection among sexual partners of infected individuals varied between 0 and 1.2% per year [26]–[27]. However, for sexual transmission to explain the persistence of HCV in Africa before the introduction of medical technologies in the 20^th^ century, one has to postulate a transmission efficacy to HCV-seronegative sexual partners substantially higher than 1% per year, otherwise the basic reproductive number (R_0_) would be much lower than unity. Sexually transmitted infections, especially those causing genital ulcerations, may enhance the efficacy of HCV transmission [22], [28]. Studies of heterosexual transmission were done in countries where genotype 2 is uncommon; the efficacy of sexual transmission may vary between genotypes or according to genetically determined factors of susceptibility.

HCV infection varied substantially between ethnic groups which could be clustered into low-prevalence and high-prevalence categories, with a 3-fold variation in prevalence. We speculate that this might relate to HCV transmission within relatively compartmentalized sexual networks. Even in a multiethnic city like Bissau, individuals (or their families) tend to select spouses within their own ethnic group. The Fulas and Mandinkas (low HCV prevalence) are predominantly Muslims, while the others are mostly animists. The Papels, Mancanhas and Mandjakos are close to each other linguistically and culturally.

Within the same population, which we had targeted to study risk factors that contributed to the build-up of the HIV-2 epidemic decades ago, HIV-2 infection was associated with past treatment for African trypanosomiasis or tuberculosis or having undergone ritual clitoridectomy, but not with transactional sex or sexually transmitted infections, suggesting that most of the HIV-2 transmission had been parenteral [18]. This may explain why HCV and HIV-2 infections were not associated.

Why was HCV prevalence, even after adjustment for ethnicity and other independent risk factors, substantially higher in individuals originating from the Biombo, Cacheu and Oio regions remains unclear. Although this must be interpreted with caution given that this subgroup was defined *post-hoc*, the very high prevalence among individuals from Bula, Encheia and Binar, three cities located within 25 km of each other, suggest that transmission occurred locally through some medical or ritual intervention, probably in the late 1940s or in the 1950s, given dates of birth and of migration to Bissau. This procedure was not excision: only 9% of women from Bula-Encheia-Binar were excised, and stratified analyses showed a much higher prevalence of HCV in those from Bula-Encheia-Binar regardless of excision status. Furthermore, men from that region were also more likely to be HCV positive. We found no association between HCV infection and having been treated for trypanosomiasis or tuberculosis (which required multiple injections), past transfusions, vaccinations, injections of pentamidine to prevent trypanosomiasis, or traditional scarifications. We then reviewed extensively the Portuguese colonial literature to look for other potential modes of parenteral transmission. Parenteral drugs were not used on a large scale for the treatment of schistosomiasis, a minor problem in the country [29]. Campaigns were organized to control yaws, but most individuals received a single intramuscular injection of long-acting penicillin [30]. Injectable contraceptives, associated with HCV in Tanzania, were probably little used in this elderly population [31]. However, we belatedly found out about another medical intervention which might have facilitated the transmission of blood-borne viruses and deserves to be investigated in future studies. When drugs active against *Mycobacterium leprae* became available (≈1955), mass treatment of leprosy patients was organized. Leprosy is not fatal and untreated cases had been accumulating for decades, so that between 1956 and 1962 treatment was initiated in 18512 leprosy patients (10113 females), ≈4% of the whole population of Portuguese Guinea [32]. In some regions, including the Bula area, leprosy patients were treated with an injectable long-acting sulfone (Hygrathione®), given intramuscularly every 2 weeks by mobile teams to patients queuing on the side of the road, during a few years for cases of tuberculoid leprosy and indefinitely for those with lepromatous leprosy [33]. This regimen, used in other countries, may explain the high prevalence of HCV among leprosy patients in Congo, Ivory Coast, Ethiopia, Yemen, Japan and Brazil [34]–[37].

Unfortunately, we had collected no information on exposure to anti-leprosy drugs. Other study limitations included the anonymous approach, which precluded linking spouses for potential HCV concordance. We sampled only individuals aged ≥50 y, and could not evaluate whether there might have been a cohort effect.

In conclusion, in Guinea-Bissau, genotype 2 HCV has probably been transmitted sexually for at least a few hundred years, and may have been more recently amplified by parenteral transmission in some specific subpopulations with the development of public health interventions. Future research should investigate the role of leprosy treatment in the parenteral transmission of HCV, as well as sexual transmission of genotype 2 HCV within couples.
